# Is Hay for the Birds? Investigating Landowner Willingness to Time Hay Harvests for Grassland Bird Conservation

**DOI:** 10.3390/ani11041030

**Published:** 2021-04-05

**Authors:** Matthew P. Gruntorad, Katherine A. Graham, Nico Arcilla, Christopher J. Chizinski

**Affiliations:** 1 School of Natural Resources, University of Nebraska-Lincoln, Lincoln, NE 68583, USA; mgruntorad2@unl.edu (M.P.G.); katherine.graham@huskers.unl.edu (K.A.G.); cchizinski2@unl.edu (C.J.C.); 2 International Bird Conservation Partnership, S-14142 Huddinge, Sweden; 3 Center for Great Plains Studies, University of Nebraska-Lincoln, Lincoln, NE 68588, USA

**Keywords:** agricultural management practices, forage crops, game birds, grassland songbirds, hay production, livestock husbandry, wildlife knowledge, hunting, conservation

## Abstract

**Simple Summary:**

Grassland and farmland bird populations are steeply declining worldwide, and conservationists are searching for solutions to prevent their continued losses. Most of these bird populations nest and raise chicks on privately owned land rather than in public protected areas. Thus, to be widely effective, conservation strategies need to engage private landowners. One promising strategy to protect grassland and farmland breeding birds is to avoid harvesting hay during the main bird breeding season, and delay hay harvest until at least 15 July, in order to allow birds to successfully nest and raise young. However, little is known about the willingness of private landowners to alter their hay harvesting practices in order to support bird conservation. We surveyed private landowners with hay production operations in the North American Great Plains to learn whether they were willing to time their hay harvests for bird conservation, and whether livestock ownership, wildlife knowledge, and hunting activity affected landowners’ willingness to time hay harvests for either songbird or game bird conservation, or both. Most respondents expressed willingness to delay hay harvesting for bird conservation. Livestock ownership and wildlife knowledge were positively correlated and hunting activity was negatively correlated with landowners’ willingness to delay hay harvest for bird conservation.

**Abstract:**

Birds in agricultural environments have exhibited steep global population declines in recent decades, and effective conservation strategies targeting their populations are urgently needed. In grasslands used for hay production, breeding birds’ nest success improves substantially if hay harvests are delayed until after mid-July. However, few studies have investigated private hay producers’ willingness to alter their harvesting practices, which is a critical factor for bird conservation where most land is privately owned, such as in the North American Great Plains. We surveyed Nebraska hay producers to examine whether livestock production, wildlife knowledge, and hunting activity affects their willingness to alter haying practices for bird conservation. The majority (60%) of respondents expressed willingness to delay harvesting hay to allow birds time to nest successfully. Livestock producers and those more knowledgeable about wildlife were more willing to delay hay harvests, whereas active hunters were less willing to do so. Our findings suggest that a majority of private producers show a high potential for engaging in grassland bird conservation activities. Landowners’ willingness to participate in bird conservation programs and actions could be further encouraged through extension and education efforts connecting hay producers with information, support, and funding for bird conservation.

## 1. Introduction

Agricultural expansion and intensification have contributed to wildlife declines worldwide [1,2,3,4], including that of 60% of the bird species on the International Union for Conservation of Nature red list [5,6]. Birds in agricultural environments, particularly species that breed in grasslands and farmlands, have exhibited steep population declines in recent decades [7,8,9,10]. In North America, for example, grassland bird populations have decreased by 50% in the past 50 years [11], and in the central Great Plains state of Nebraska, 60% of breeding bird species are declining [12]. Conservation actions targeting grassland and farmland bird populations are urgently needed. In agricultural environments in particular, where most land is privately owned, engaging private landowners in bird conservation is critical for mitigating ongoing bird declines [13]. However, little attention has been given to the importance of conservation on private lands, as well as the role of landowners, compared with public lands and protected areas [14].

In grasslands and meadows used for hay production, harvesting hay during the bird breeding season destroys nests and causes the mortality of incubating females, which has contributed to grassland bird population declines [15,16,17,18,19]. By contrast, timing hay harvests to occur outside the main bird breeding season delivers measurable benefits for both songbird and game bird conservation [15,20,21,22,23,24,25,26]. Waiting until mid-July or later to mow fields allows breeding birds to complete at least one nesting cycle [27] and delivers measurable improvements in bird and nest densities, nest success and recruitment, and annual return rates for both songbirds and game birds [24,28,29,30]. Thus, many conservation programs encourage farming and ranching operations to delay hay harvest until at least 15 July [31]. However, a delayed hay harvest may result in the loss of nutritional quality [26] and may also reduce the crop’s monetary value and create additional labor costs for landowners [24].

There is an extensive body of literature on incentives to support wildlife conservation management on private land [32,33,34], and a number of studies have documented the willingness and ability of landowners to alter their management practices to benefit specific bird species [35]. Studies of American landowners’ engagement with the US Endangered Species Act have suggested that many landowners make willing and valuable conservation partners [36]. Furthermore, studies in the northeastern United States have documented landowners’ willingness to improve habitat quality when they believe management actions will result in positive conservation outcomes, or when motivated to use the property for hunting activities [37,38,39]. Troy et al. [38] indicated that approximately half of Vermont dairy farmers surveyed were willing to change their haying practices to allow songbirds sufficient time to nest and raise young on at least a portion of their property. Harvest management strategies in Vermont were met with success when farmers harvested hay early (late May-early June) and waited 65 days before harvesting again to allow birds the opportunity to re-nest [31]. However, early hay harvest strategies may be complicated by environmental constraints in other regions [16,40].

When making land use decisions, agricultural producers draw from both a business context, in which extrinsic drivers influence decision making [41,42,43], and an intrinsic personal context [44]. In Vermont [38], many hay producers are engaged in dairy operations, whereas by contrast many prairie-meadow hay producers in Nebraska incorporate beef cattle in their ranching operations or sell their hay crop to beef producers to sustain their cattle herds through the winter months. Research focused on beef producers’ adoption of conservation practices is limited [45]. In the Nebraska Sandhills, wet prairie-meadow hay harvest, hereafter referred to as harvest, occurs from late June to early August [46]. Because of the economical and nutritional constraints ranchers face for altering their haying practices, understanding their willingness and ability to delay harvest is critical to future conservation planning efforts. In the Nebraska Sandhills, harvest in late June results in higher quality hay, but is often not possible because of wet or inundated soil, while hay harvested in the latter portion of the season is low in forage quality and may not provide enough nutrient content for spring-calving cows [47]. During this period (late June), many grassland bird species are also nesting, incubating eggs, and raising chicks [18,20,48].

Here, we conducted a survey of Nebraska Sandhills landowners, and examined how extrinsic (livestock ownership) and intrinsic (wildlife knowledge and hunting activity) factors influenced a conservation action (willingness to delay harvest). Our objectives were to understand current hay management practices and landowners’ willingness to alter management strategies in ways that benefit grassland bird conservation; to compare the willingness of landowners to alter management strategies for the purpose of game and non-game bird nesting success; and to learn whether hay producers’ willingness to time hay harvests for bird conservation was positively or negatively correlated with livestock ownership, wildlife knowledge, and/or hunting activity.

We drew from value–belief–norm theory (VBN) as the basis for our study, with the argument that specific environmental-value orientations inform individuals’ beliefs and personal norms, which ultimately determine their proclivity for pro-environmental behavior [49,50]. Currently, three environmental value orientations have been described, namely, intrinsic, instrumental, and relational [51,52,53,54], which are used as a means to describe relationships between humans and nature. Briefly, the intrinsic orientation is described as a value of nature for what it is and not what it provides [55]. Instrumental values pertain to the value of the environment for a particular end, such as the value of prairie meadows to produce hay and feed livestock in a ranching operation. Relational values are a relatively new topic of discussion and are focused on an individual’s relationship with nature [56,57]. Specifically, relational values are centered around expressions of care and concern for the environment [52,58]. The value–belief–norm theory has been used successfully to explain a variety of general pro-environmental behaviors [59,60]. In this context, we predict that increased wildlife knowledge, livestock production, and hunting participation are tied to environmental value orientations and may influence willingness to delay hay harvest.

We hypothesize that landowners might be more inclined to delay haying to benefit game bird conservation than songbird conservation, based on the fact that game birds, such as prairie-chickens, pheasants, and ducks, may be more easily recognized by the public and have been subjects of regional conservation initiatives. We also hypothesize that livestock ownership will be positively correlated with hay producers’ willingness to time harvests for bird conservation, as previous research has suggested that landowners with farming operations incorporating livestock are more likely to participate in conservation practices [61,62,63], potentially because of lifestyle aspects affiliated with farming [42,64,65]. Hay producers who also produce beef may therefore be more likely to delay harvest than operators who do not maintain livestock. Intrinsic factors influencing land use decisions have been reflected in personal capabilities, such as motivations, attitudes, and knowledge [66,67,68,69].

Knowledge and awareness of natural resources can also affect intrinsically driven land use decisions and conservation practices. For example, research has demonstrated that ranchers who are aware of the importance of carbon sequestration are more likely to implement improved land management practices that increase soil carbon levels [70]. Thus, we hypothesize that wildlife knowledge, of both game and non-game species inhabiting prairie meadows, would positively affect landowner willingness to delay hay harvests for bird conservation. Many landowners in this region pursue recreational hunting activities, as the portion of Nebraska Sandhills in our study has a number of game species including waterfowl, upland birds, white-tailed deer (*Odocoileus virginianus*), and wild turkey (*Meleagris gallopavo*). Thus, we also hypothesize that landowners engaged in hunting activities may be more willing to delay hay harvest to manage and conserve game species of interest, if not for the sake of non-game grassland bird populations.

## 2. Materials and Methods

### 2.1. Study Area and Background

**Figure 1:**
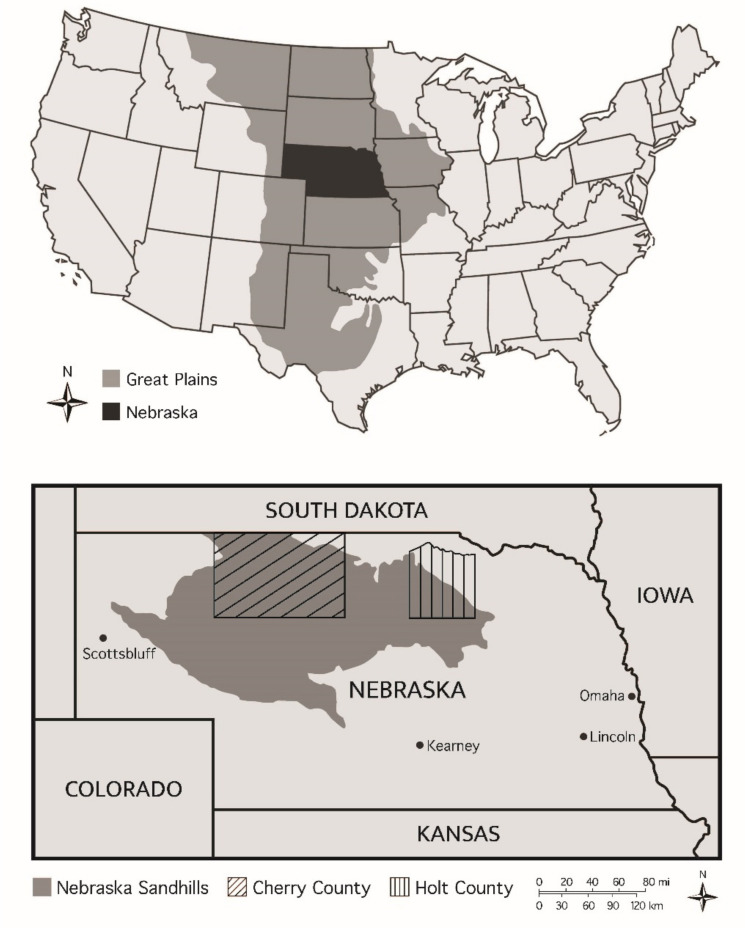
Map of the study area in the North American Great Plains.

For our study area we selected Holt and Cherry counties (Figure 1), the two most productive Nebraska counties for non-alfalfa hay production, which combined produced over 8 million tons of hay in 2017 (see Supplementary Table S1 for Holt and Cherry counties hay statistics). The Sandhills region of Nebraska covers more than 5.2 million hectares of largely contiguous grassland in the north-central portion of the state [71,72]. The largest intact grassland remaining in the North American Great Plains [73], this area of “mixed” prairie, is almost entirely privately owned and used mainly as rangeland [74,75,76]. About 10% of the Sandhills region comprises wet meadows of cool-season grasses (e.g., *Hesperostipa comate*, *Koeleria macrantha*, and *Phalaris arundinacea*), legumes, and native sedge and rush species primarily used as a source of hay for feeding cattle when prairie vegetation becomes dormant [46,77].

### 2.2. Survey Methods and Instruments

We developed a draft questionnaire in the summer of 2018 and reached out to a number of groups for suggestions to improve questionnaire clarity and pertinence to hay producers. We incorporated suggestions from university extension agents, wildlife agency biologists, and ranchers from the Sandhills region into the questionnaire. A final version of the questionnaire was mailed in February 2019 to 823 rural property owners in the Holt and Cherry counties (see Supplementary Table S2 for survey questionnaire). We developed our sampling framework using a combination of publicly available land parcel ownership information and plot maps containing vegetation information. We exclusively selected land parcels containing what appeared to be wet prairie meadow in our sampling frame. We included a cover letter detailing the purpose of the study along with a questionnaire and a postage-paid return envelope in each mailing. A reminder letter and replacement questionnaire were mailed to all non-respondents four weeks after the initial mailing. We closed the survey when responses stopped coming in late March, about eight weeks after the initial mailing. Participants were informed that, by submitting their completed questionnaire, they were consenting to participate in the study. The University of Nebraska-Lincoln Institutional Review Board approved all protocol and survey instruments (IRB approval no. 20190119057 EX).

The first section of the questionnaire included questions pertaining to aspects of the ranching operation, such as whether or not the operation included livestock production or incorporated wet prairie-meadow hay production. If respondents indicated that they harvested hay, they were additionally asked when harvest typically began and when harvest was typically completed in two open-ended questions. Section two contained the wildlife knowledge portion of the questionnaire. This knowledge section consisted of four sets of yes/no questions, with each set relating to a separate bird species. Two sets of questions were related to game bird species, mallard duck (*Anas platyrhynchos*) and greater prairie-chicken (*Tympanuchus cupido*), two conspicuous and easily recognized game bird species in the area. The other two question sets focused on songbird species, western meadowlark (*Sturnella neglecta*) and bobolink (*Dolichonyx oryzivorus*), two conspicuous and easily recognized songbird species in the area.

Each wildlife knowledge question set consisted of the following five questions: whether or not the respondent could identify the species, whether or not the species was found in Nebraska year-round, whether or not the species breeds in Nebraska, whether or not the species needs prairie meadows to nest and raise young, and whether or not haying prairie meadows affects nesting success of the species. We conferred with agency biologists and produced an accurate answer key for scoring the knowledge section of the questionnaire (see Supplementary Table S3 for answer key of wildlife knowledge section of the questionnaire). Section 3 included a question about what type of hunting activities the respondent participated in, and respondents had the option to select any combination of big game, upland game, turkey, and waterfowl, or alternatively, “I do not hunt”. The final section included two questions about their willingness to delay harvest until after mid-July. On a five-point scale (ranging from very unlikely to very likely), respondents were asked how likely they were willing to delay at least a portion of their harvest for the purpose of songbird conservation, and in a separate question, their willingness to delay for the purpose of game bird conservation.

### 2.3. Statistical Analyses

Non-response to questionnaire surveys may potentially bias results, as those who respond to such questionnaires may differ in some systematic way from non-respondents [78,79,80]. To estimate non-response bias, we compared the responses from the initial invitation to the responses we received after sending the reminder mailing. The use of the second or final wave to measure non-response bias reflects the extrapolation methods, which are based on the assumption that individuals who respond after reminders are more likely to be similar to non-respondents [81,82].

We used ordinal regression to compare willingness to delay hay harvest as the dependent variable and the conservation purpose (songbird or game bird) as the predictor variable [83]. We tested two additional ordinal regression models to assess whether the covariates of wildlife knowledge, livestock production, and hunting activity have an effect on producers’ willingness to delay harvest for songbird conservation and game bird conservation. Wildlife knowledge was assessed as a continuous variable represented by the total number of wildlife knowledge questions respondents answered correctly, with possible values ranging from 0 to 28. Livestock production was measured as a binary variable with respondents either indicating they did or did not produce livestock. Hunting activity was assessed as a continuous variable ranging from 0 (did not hunt) to 4 (participated in big game, upland game, waterfowl, and turkey hunting). We conducted all modeling using the package ordinal [84] in R [85]. We presented the likelihood of a covariate affecting willingness of producers to delay harvest as the odds ratio (OR), calculated using the lsmeans package [86] in R. We used Cramér’s V (φc) to measure the effect size. We conducted imputations for missing values in willingness to delay harvest using the “proportional odds” (polr) method, with harvest start month and harvest end month as predictors in the mice package in R [87].

## 3. Results

The response rate for the survey was approximately 36% (294 returned surveys). Item non-response reduced the sample size to 229. We collected responses from 151 respondents prior to our reminder mailing and responses from 78 respondents after the reminder mailing. We concluded that the general non-response bias was small, as there were no significant differences (*p* > 0.05 level) in likelihood to delay harvest for either songbirds or game birds, probability of producing livestock, wildlife knowledge, or hunting activity between those who responded to the initial invitation and those who responded to the reminder mailing. Because of the similarity between early and late respondents in our measures and no indication of non-response bias, the later responses to the survey were included in the analyses [88].

### 3.1. Descriptive Statistics

**Figure 2:**
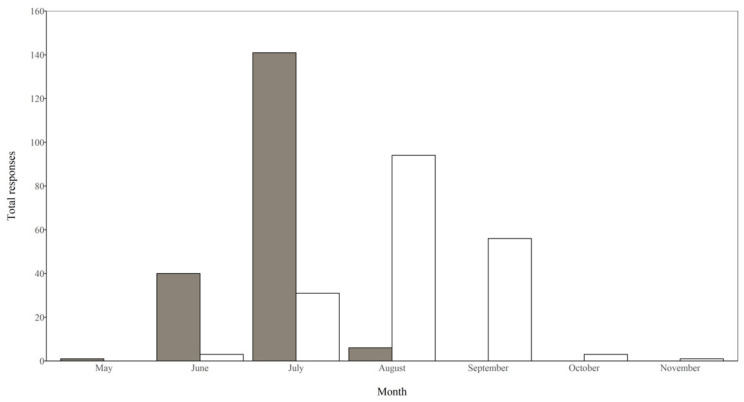
Typical start (shaded) and finish (open) months of prairie hay harvest by ranchers in the Holt and Cherry counties, Nebraska, USA surveyed in 2019.

The specificity of responses as to when ranchers typically began harvesting prairie hay varied from vague generalizations (“when it is dry”) to specific calendar dates (“1 June”). Some respondents answered with only the name of a month, while other respondents offered a specific week or set of weeks (“first or second week in July”). We combined the responses by month. The majority of ranching operations began hay harvest in the month of July (Figure 2). Similar to responses regarding harvest start time, the specificity of responses to harvest end varied. We combined the responses by month, with most respondents finishing hay harvest in the month of August. Overall, most respondents were either likely or very likely to delay harvest for the conservation of both songbirds and game birds (Table 1).

Most respondents indicated that their ranching operation incorporated the harvest of prairie meadows and livestock production (Table 1). Respondents, on average, participated in only one type of hunting activity and were more likely to not participate in any hunting than in any one particular type. Of those who did partake in hunting activity, big-game hunting was the most practiced (Table 1). Respondent knowledge of game bird species was relatively greater than knowledge about non-game birds for both hunters and non-hunters. On average, respondents had relatively little knowledge about bobolink compared with mallard duck, greater prairie-chicken, and western meadowlark (Table 1).

### 3.2. Factors Associated with Willingness to Delay Harvest

**Figure 3:**
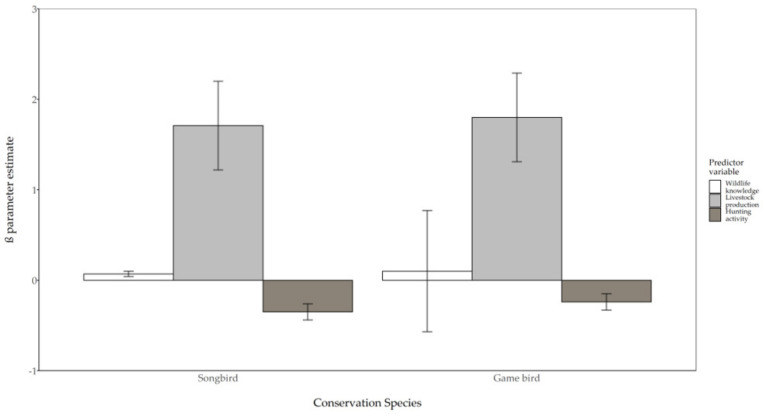
Effects of wildlife knowledge (white bars), livestock production (light gray bars), and hunting activity (dark gray bars) on likelihood to delay prairie hay harvest for the purposes of songbird and game bird conservation by ranchers in the Holt and Cherry counties, Nebraska, USA, 2019. β parameter estimates with standard errors for songbird conservation depict directional transition from little to great wildlife knowledge (χ^2^ = 7.60; odds ratio = 1.08; 95% CI = 1.02–1.14; *p* < 0.01; Cramér’s V = 0.34), non-livestock producers to livestock producers (χ^2^ = 12.10; odds ratio = 5.53; 95% CI = 2.11–14.50; *p* < 0.01; Cramér’s V = 0.27), and no hunting activity to multiple hunting activities (χ^2^ = 14.89; Odds ratio = 0.71; 95% CI = 0.59–0.84; *p* < 0.01; Cramér’s V = 0.18). β parameter estimates with standard errors for game bird conservation depict directional transition from little to great wildlife knowledge (χ^2^ = 13.01; odds ratio = 1.11; 95% CI = 1.05–1.17; *p* < 0.01; Cramér’s V = 0.36), non-livestock producers to livestock producers (χ^2^ = 13.29; odds ratio = 6.05; 95% CI = 2.31–15.86; *p* < 0.01; Cramér’s V = 0.27), and no hunting activity to multiple hunting activities (χ^2^ = 6.91; odds ratio = 0.78; 95% CI = 0.65–0.94; *p* < 0.01; Cramér’s V = 0.18).

We found that producers were equally likely to delay harvest for the conservation of songbirds as for game birds (χ^2^ = 0.03, *p* = 0.86). Livestock production, wildlife knowledge, and hunting activity all affected the likelihood of hay producers’ willingness to delay harvest for songbird conservation (model χ^2^ = 26.75, *p* < 0.01) and game bird conservation (model χ^2^ = 25.79, *p* < 0.01), although the directions of these relationships varied. Whether or not ranching operations incorporated livestock production had the greatest effect on likelihood to delay harvest for both songbirds and game birds, with livestock producers more likely to delay than hay producers who did not produce livestock (Figure 3). Similarly, respondents who possessed greater knowledge of wildlife were more likely to delay harvest to conserve both groups of bird species. Hunting activity had a negative association with willingness to delay harvest.

## 4. Discussion

Our results suggest that a majority of landowners are willing to delay hay harvest for grassland breeding bird conservation. Specifically, 60% of respondents reported that they were either likely or very likely to delay harvest for the conservation of both songbirds and game birds. This first evidence that the delayed harvest of wet prairie meadows may be a feasible practice for hay producers in the North American Great Plains is highly encouraging for advancing grassland bird conservation in this region, where 98% of the land is privately-owned [9,89]. These findings are consistent with previous research on American landowners’ engagement with the US Endangered Species Act, which suggested that many make willing and valuable conservation partners [36]. Our results also compare favorably with the only similar study to ours that we found in the peer-reviewed literature [38], in which approximately half of Vermont dairy farmers surveyed were willing to change their haying practices to allow songbirds sufficient time to nest and raise young on at least a portion of their property, especially among farmers with smaller cattle herds. Likewise, a survey in Kenyan grasslands found nearly half (44%) of farmers expressed a willingness to improve the area’s conservation value [90].

The current timing of haying (July–August) in the Nebraska Sandhills occurs after peak western meadowlark initial nesting frequency during early to mid-May, allowing sufficient time (~50 days) for juveniles fledged from initial nests to fly well enough to avoid destruction by haying operations [91,92]. During this period of harvest, many grassland bird species are also incubating or nesting [18,20,48]. This timing is favorable to “early bird” nesting success (first attempts of early nesting birds), but further delaying hay harvests would allow for additional nesting attempts and time for nestlings and fledglings to develop mobility [92], further increasing nest success and juvenile survival of more individuals and species. In Nova Scotia, for example, delaying hay harvest by 1.5 weeks (from 20 June to 1 July) increased songbird fledging rates up to 56%, and postponing hay harvest for one additional week (to 7 July) enabled songbirds to maximize their fledging rates [24].

In the Nebraska Sandhills, songbirds, in addition to meadowlarks and bobolinks, that may benefit from delayed haying include cassin’s sparrows (*Peucaea cassinii*), lark sparrows (*Chondestes grammacus*), and grasshopper sparrows (*Ammodramus savannarum*), which are reported as having active nests in the Nebraska Sandhills through at least mid-July [93]. In addition, game birds in this region, including sharp-tailed grouse (*Tympanuchus phasianellus*), have active nests through at least late June [94], and greater prairie-chickens have active nests through at least early July [95]. All of these species, as well as others that have not been studied in this region, would likely benefit from delayed haying until at least 15 July, as recommended in numerous studies of grassland bird nesting success, as nests and young fledgling do not survive haying operations [15,20,21,22,23,24,25,26].

Nebraska hay producers expressed equal willingness to delay harvest for the conservation of songbirds as for game birds, although on average, the producers’ knowledge of game birds was greater than their knowledge of songbirds. This finding suggests that songbird education targeting landowners in this area may successfully bolster bird conservation because landowners are willing to protect songbirds but may lack information and support to build this willingness into conservation action. Our findings also agree with studies in the northeastern United States that have documented landowners’ willingness to improve habitat quality when they believe management actions will result in positive conservation outcomes, or when motivated to use the property for hunting activities [37,39].

Encouraging and expanding grassland bird conservation on private lands will require giving attention to landowners’ conservation interests and motivations. Participants in grassland bird conservation programs in the northeastern United States, for example, have reported being motivated by a deep interest in environmental issues, and birds in particular, to support hay producers’ protection of nesting birds [96]. In the central and western United States, the National Audubon Society’s Conservation Ranching Program provides an example of a program that certifies “bird-friendly” ranching practices, including engaging Texas ranchers in agreements to avoid harvesting native grassland for hay during the main bird breeding season [97]. A survey of Dutch farmers’ willingness to reduce pesticide use found that respondents were motivated by environmental considerations and also strongly influenced by other farmers’ decisions in this area [98]. Likewise, a survey elsewhere in the Great Plains that investigated landowners’ willingness to reduce non-native grasses on their land showed that respondents were motivated by a sense of moral responsibility, as well as by social considerations [99].

Our results follow the VBN theory, which suggests that specific environmental-value orientations ultimately determine proclivity for pro-environmental behavior. Consistent with previous research, we found that livestock production had a positive effect on willingness to adopt conservation practices [61,62,63], suggesting that livestock producers in the Nebraska Sandhills would be good targets in the future for both game and non-game conservation programs. This is encouraging, as the majority of producers in our study incorporate livestock in their operations. Moreover, livestock production had the greatest effect on likelihood to delay harvest for both songbirds and game birds, although it should be noted that only 22 respondents indicated that they do not have livestock in their operation. This finding contributes to a sparse body of current knowledge on beef producers’ adoption of conservation practices. Hay harvested later during the bird breeding season is lower in forage quality and may not contribute sufficient nutrient content for spring-calving cows [47]. Despite this, livestock producers professed a willingness to delay hay harvest for conservation, indicating that they may in part utilize an intrinsic environmental value orientation to shape their farming practices, rather than identifying solely with an instrumental orientation and prioritizing economic gain.

Additionally, we found that greater wildlife knowledge resulted in increased willingness to delay hay cutting, for the conservation of both songbirds and game birds. Encouragingly, producers were generally knowledgeable about the majority of bird species in our questionnaire. From the comments section of our questionnaire, it was clear that many of the families in the Nebraska Sandhills have a long history of agriculture production on their land, spanning multiple generations. Several comments about appreciation for wildlife on their land also support our results about wildlife knowledge and suggest the community is generally well connected to the land they operate and the wildlife that reside on it. Among the four species included in our questionnaire, respondents possessed the least knowledge about the bobolink. The relative lack of knowledge possessed by area producers is concerning, as bobolinks have declined by 60% since 1970 [10,100], and low reproductive success has largely driven their population decline [101].

Although studies in Europe have documented benefits to declining grassland bird populations from land management practices tailored to promote hunting wildlife [102,103,104], we found a negative correlation between hunting activity and willingness to delay harvest for bird conservation. Most respondents engaged in hunting reported involvement in big game hunting, such as white-tailed deer. One possible reason for this may be hunters’ motivation to begin their harvest earlier in the season to allow more time to hunt during the fall hunting seasons. Hunters may also wish to harvest prairie meadows earlier in the season to encourage earlier regrowth of prairie meadow vegetation, which may enhance cover and habitat quality for game species during the hunting season, or provide more dense cover in the winter [105,106,107].

In our study area, the majority of ranching operations did not begin hay harvest until the month of July (Figure 2). We hypothesize that the timing of hay cutting in this region may be partly due to accessibility (i.e., it is too wet until July). Diemer and Nocera [16] reported that over the past 50 years in southern Ontario, haying has shifted to take place 14–21 days earlier because of earlier maturing grass species, increased mechanization, and more frequent haying. A monetary plan to encourage delayed hay harvest may need to be considered in the future if similar trends emerge in the Great Plains region.

Where suitable, landowners could be connected with funding and financial incentives supporting bird conservation. For example, the U. S. Farm Bill, a federal government program, pays farmers for wildlife conservation practices as a public good, such as through the Conservation Reserve Program that supports the conservation of 24 million acres of private land through 10-year contracts (Askins, 2002; Powell, 2019). Wildlife conservation incentives were added to the Farm Bill in 1986 [9], since which the Natural Resources Conservation Service has provided competitive awards totaling $360 million to public–private environmental partnerships, including hay producers willing to delay haying to allow grassland birds to nest successfully [96,108]. The Conservation Reserve Program [7,9] has aided in grassland bird population changes from −34% prior to 1985 to +3% since 2003 [9,89].

## 5. Conclusions

Although delaying hay harvest for bird conservation may have wider geographical applications, its regional feasibility requires specific knowledge of local agricultural and biological differences [16,40]. The longstanding land ethic tradition present in the Nebraska Sandhills, in which environmental stewardship has been practiced for generations, is likely related to respondents’ expressed willingness to delay hay harvest to support bird conservation. Engaging these respondents and like-minded hay-producers through follow-up guidance and information regarding bird-friendly grassland management practices may encourage action for bird conservation [109]. Encouraging such practices on a regional scale will also require engagement with producers to evaluate incentives and understand any perceived barriers [109,110,111,112].

Many farmers and ranchers with positive perceptions of and attitudes toward birds and other wildlife have reported that their ability to protect bird habitats was limited by financial constraints and that subsidies would improve their capacity to deliver conservation services [109,110,111,112]. We hypothesize that economic constraints may play a role for hay producers otherwise not willing or able to delay hay cutting in Nebraska. In such cases, direct payments from targeted conservation programs may offset financial losses for private landowners who are otherwise unable to afford the associated costs of lower hay quality resulting from delayed haying [113]. Given the expressed willingness of a majority of respondents to delay hay harvest to benefit bird conservation, a targeted conservation scheme could be developed to engage producers in Nebraska, and the larger Great Plains region. For this to happen, the consultation and exchange of information with producers to consider possible approaches and actions [114,115] will be necessary.

As one of North America’s largest remaining contiguous prairie systems, the Nebraska Sandhills is likely a major population source for grassland bird species limited to fragmented and marginal habitats elsewhere [92]. Improving bird conservation activities in this region therefore has the potential to significantly improve net recruitment into many grassland breeding bird populations that are currently declining or stable [116]. Both voluntary schemes and those that include direct payments to offset financial costs have been implemented successfully to assist producers in other regions [113]. During the first year of The Bobolink Project (2013), 210 donors gave $32,000 to support hay producers’ delayed harvest on 200 acres of grassland; in the second year of the project, the number of participating landowners tripled [117]. Participating producers reported noticing a positive difference in the number of birds returning and that the project enhanced their appreciation of birds and nature. They also reported wanting to use the project to teach their children awareness of human relationships with nature, and specifically to give back as well as take from nature [96].

That a majority of respondents expressed willingness to time hay harvests for bird conservation is likely due to not only accessibility reasons, but is also associated with the fact that a large proportion of hay producers in this area incorporate livestock in their operations and are quite knowledgeable about the wildlife in the area. Outreach efforts to the hunting community may promote greater nesting success of songbird species. Additionally, a program focused on increasing wildlife knowledge, specifically songbird knowledge, may result in further willingness to participate in wildlife conservation programs. We suggest future directions include following up on this survey by reaching out to respondents who expressed willingness to time hay harvests for bird conservation and connecting them with information and support regarding funding already available to do so through Farm Bill and other relevant programs [118].

Education about the efforts of prairie-hay cutting and the timeframe of songbird and game bird nesting activity may stimulate a regional awareness in the same way outreach programs in similar areas have garnered in previous research [119,120]. In New England, where haying during the bird breeding season is considered the leading threat to grassland-nesting birds, adjusting haying schedules is recommended as the most effective action to assist their recovery, both held in the public trust and by municipalities, land trusts, and conservation groups [109]. Through targeted outreach and engagement with land managers, adopting bird-friendly haying schedules may achieve significant improvements in the conservation status of increasingly imperiled grassland-nesting birds. In the same way, our findings highlight the feasibility of a way forward to deliver significant improvements in grassland bird conservation, both in the Nebraska Sandhills and elsewhere.

## Figures and Tables

**Table 1:** Summary statistics for hay harvest, livestock production, wildlife knowledge, and hunting activity from landowners in Nebraska, USA, 2019. ^1^

Survey Variable	$\%\;\mathrm{or}\;\bar{x}$	SE
Harvest prairie meadows (yes)	91.0	
Produce livestock (yes)	90.3	
Harvest prairie meadows (yes) and produce livestock (yes)	82.0	
Hunting activity $\bar{x}$	1.0	0.10
Big game	21.0	
Upland game	19.0	
Turkey	11.8	
Waterfowl	11.8	
None	36.4	
Wildlife knowledge $\bar{x}$ (0–24)	15.3	0.37
Mallard duck $\bar{x}$ (0–6)	4.1	0.11
Greater prairie chicken $\bar{x}$ (0–6)	4.8	0.12
Western meadowlark $\bar{x}$ (0–6)	4.3	0.12
Bobolink $\bar{x}$ (0–6)	2.1	0.16
Delay harvest for songbirds		
Very unlikely	12.6	
Unlikely	11.5	
Neutral	16.2	
Likely	24.1	
Very likely	35.6	
Delay harvest for game birds		
Very unlikely	11.7	
Unlikely	9.6	
Neutral	18.1	
Likely	30.3	
Very likely	30.3	

^1^ Ranchers in the Holt and Cherry counties were surveyed in February 2019 regarding willingness to delay hay harvest until mid-July for songbird and game bird conservation.

## Data Availability

Data supporting reported results can be obtained from the authors.

## Supplementary Materials

The following are available online at https://www.mdpi.com/article/10.3390/ani11041030/s1. Table S1. Holt and Cherry counties hay statistics in 2017. Table S2. Survey questionnaire. Table S3. Answer key for the wildlife knowledge section of the questionnaire.
